# Hospital-Based Program to Increase Child Safety Restraint Use among Birthing Mothers in China

**DOI:** 10.1371/journal.pone.0105100

**Published:** 2014-08-18

**Authors:** Xiaojun Chen, Jingzhen Yang, Corinne Peek-Asa, Kangwen Chen, Xiangxiang Liu, Liping Li

**Affiliations:** 1 The First Affiliated Hospital of Shantou University Medical College, Shantou, China; 2 Injury Prevention Research Center, Shantou University Medical College, Shantou, China; 3 Injury Prevention Research Center, The University of Iowa, Iowa City, Iowa, United States of America; 4 The Women and Children Hospital of Shantou, Shantou, China; CUNY, United States of America

## Abstract

**Objective:**

To evaluate a hospital-based educational program to increase child safety restraint knowledge and use among birthing mothers.

**Methods:**

A prospective experimental and control study was performed in the Obstetrics department of hospitals. A total of 216 new birthing mothers from two hospitals (114 from intervention hospital and 102 from comparison hospital) were recruited and enrolled in the study. Intervention mothers received a height chart, an 8-minute video and a folded pamphlet regarding child safety restraint use during their hospital stay after giving birth. Evaluation data on the child safety seat (CSS) awareness, attitudes, and use were collected among both groups before and after the intervention. An additional phone interview was conducted among the intervention mothers two months after discharge.

**Results:**

No significant differences existed between groups when comparing demographics. Over 90% of the intervention mothers found the educational intervention to be helpful to some extent. A significantly higher percentage of mothers in the intervention than the comparison group reported that CSS are necessary and are the safest seating practice. Nearly 20% of the intervention mothers actually purchased CSS for their babies after the intervention. While in both the intervention and comparison group, over 80% of mothers identified the ages of two through five as needing CSS, fewer than 50% of both groups identified infants as needing CSS, even after the intervention.

**Conclusion:**

The results indicated that child safety restraint education implemented in hospitals helps increase birthing mothers' overall knowledge and use of CSS. Further efforts are needed to address specific age-related needs to promote car seats use among infants.

## Introduction

Road crashes are the leading cause of death and injury in children in China [1], but China has not adopted national policies for child safety restraints in cars [2]. In the absence of legislation and public education campaigns, parents' knowledge of child safety seats is poor, resulting in a very low rate of child safety seat (CSS) use in China [4]. A 2012 survey in the large city of Shanghai found that only 5.1% of children under the age of 7 were restrained while traveling in a car [3]. Our 2012 observational survey in the middle-sized city (and therefore less influenced by international trends) of Shantou found that fewer than 1% of children were restrained [4].Correct use of a child safety restraint in the proper seating position can reduce the risk of fatality by up to 71% and the risk of serious injury up to 67% [5]. With rapidly increased motorization in China, there is a great need to develop and test intervention strategies that could effectively increase child safety restraint usage in China [6], since Chinese parenting customs coupled with low safety awareness could serve as barriers to CSS use.

To ensure safety of children from birth, in the 1990s, the American Academy of Pediatrics recommended that all newborns discharged from hospitals in the United States be transported home in car safety seats that met the Federal Motor Vehicle Safety Standards and that hospitals should have comprehensive policies and procedures in place for the discharge of newborns [7]–[9]. Because of these recommendations, along with legislation requiring the use of car safety seats or child restraint devices for infants and young children, many hospitals in the United States have implemented education programs for child restraint. These programs include educating parents, regular review and revision of educational materials, and periodic in-service education for responsible staff [10]–[12]. These programs, along with the fact that many hospitals provide the safety seats to families required by law, have helped reduce injuries and deaths of children while traveling in a car [13]. Currently, there is no regulation on child safety restraint use in China, and few programs have been conducted to educate new parents about CSS so far [14]–[15].

Every day in China, about 50000 infants are born, with the vast majority of them born in hospitals. Most of these infants leave the hospital unrestrained and will never be placed in a CSS. Thus, educating parents of newborns about the importance of using a CSS is essential, and the period spent in the hospital for the birth could be the ideal setting to teach birthing mothers about CSS and motivate them to use them. This is the first report of educational intervention in the hospital setting in China. The purpose of this study was to evaluate the effectiveness of a hospital-based CSS education program on increasing parents' awareness and behavior regarding child occupant restraint use. It can help provide evidence about the potential impact.

## Methods

### Study design and participants

This study utilized a quasi-experimental pre- and post- nonequivalent control design and was conducted in two hospitals in Shantou, a city located in Eastern China, between May and September of 2013. The University-affiliated Hospital, a non-profit general hospital providing 50 ward beds with 250 babies delivered per month, served as the intervention site. The Women and Children Hospital, a non-profit general hospital providing 60 ward beds with 300 babies delivered per month, served as a comparison site. These two hospitals were selected as they were located in the same district with a similar number of ward beds for delivery. The patient populations served by the two hospitals were generally similar in terms of income and education status. Since in-hospital bed availability in China is currently limited, a ward is usually shared by 3 to 4 birthing mothers in the Obstetrics Department, and new mothers are likely to exchange information. To minimize potential contamination, the intervention and comparison group were assigned to different hospitals rather than randomized individually.

Eligible participants were mothers who delivered at the participating hospitals with a successful live birth without complications, were discharged from the hospital after the customary four to six day postpartum stay, were owners of a car, and agreed to participate in the study by signing informed consent. Participating mothers who gave birth in the Obstetric Department of the University-affiliated Hospital and met the study inclusion criteria were assigned into the intervention group. Intervention mothers received an educational intervention regarding child safety restraint during their postpartum stay. Participating mothers who gave birth in the Women and Children hospital and met the study inclusion criteria were assigned into the comparison group; These mothers were given a printed height chart and general information about how to measure their baby's height. No information on car safety restraint was provided to mothers in the comparison group during their postpartum stay.

The study and consent process was approved by Medical Ethics Committees of Shantou University Medical College.

### Procedure

The study information was distributed in both intervention and comparison hospitals to mothers on the second day after they gave birth. Mothers who expressed interest were screened for eligibility and enrolled. Baseline data collected from both intervention and comparison participants included demographics, awareness, and attitudes on child safety restraint. Following the baseline, the education intervention was delivered to the participating mothers who gave birth in the intervention hospital. A trained research staff met with the mothers individually to provide three educational sessions on child safety restraint during their hospital stay. Prior to discharge, mothers in both groups were asked to complete follow-up surveys regarding their awareness and perception of child safety restraints. Two months after discharge, mothers in the intervention group were re-contacted by telephone interview and asked about their use of child safety restraint.

### Education intervention

The intervention consisting of three education components was delivered to the birthing mothers: (1) A height chart illustrating proper age for child safety seat use, and legislation regarding CSS use in western countries was shown and explained to the new mothers by trained research staff following enrollment and the baseline survey. (2) An 8-minute video was shown on a Child Car Crash Test involving a child in the vehicle, displaying different results with or without the use of a child safety restraint, and demonstration on how to correctly use a CSS. The video was displayed via an iPad for each individual mother two days following enrollment. (3) A pamphlet on automobile crash protection and safe seating position in the car for children was handed out to the new mothers, who were asked to read it carefully. Three components of the intervention were delivered to birthing mothers in three different and consecutive days following enrollment before discharge: te height chart was given on day 1, the car crash video was watched on day 2, and the pamphlet was read on day 3.

### Main outcome measures

*CSS Awareness* was measured by 5 survey items related to child safety restraint. Participating mothers were asked about their awareness of the safest seating position for a child in a car, safe traveling practices in a car, proper age of using child safety restraint, substitute of seat belt, and classification of CSS. Data were collected before and after the intervention among the intervention and comparison groups.

*Attitudes towards CSS use* was measured by 3 questions asking about participating mothers' perceptions on the necessity of CSS use, consideration of future usage, and reasons why current CSS use was not popular in China. Data were collected before and after the intervention among the intervention and comparison groups.

*CSS use behavior* was measured by participating mothers' self-reported actual use of CSS including having purchased a CSS and type of CSS, and intention of future use.

### Statistical analysis

Descriptive statistics were used to describe the characteristics of participation mothers in both intervention and control groups. Birthing mothers' ratings on helpfulness of each intervention component were reported. The ratings for a subgroup of mothers who actually purchased or used CSS following the intervention were reported and compared to their counterparts. Differences in birthing mothers' awareness of, attitude towards, and use behaviors of child safety restraint before and after the intervention were compared between intervention and comparison groups, before and after the intervention. Chi-square tests were used to test the differences in proportions, with significance level set at α = 0.05. All analyses were conducted in SPSS 20.

## Results

### Characteristics of participating mothers

**Figure 1:**
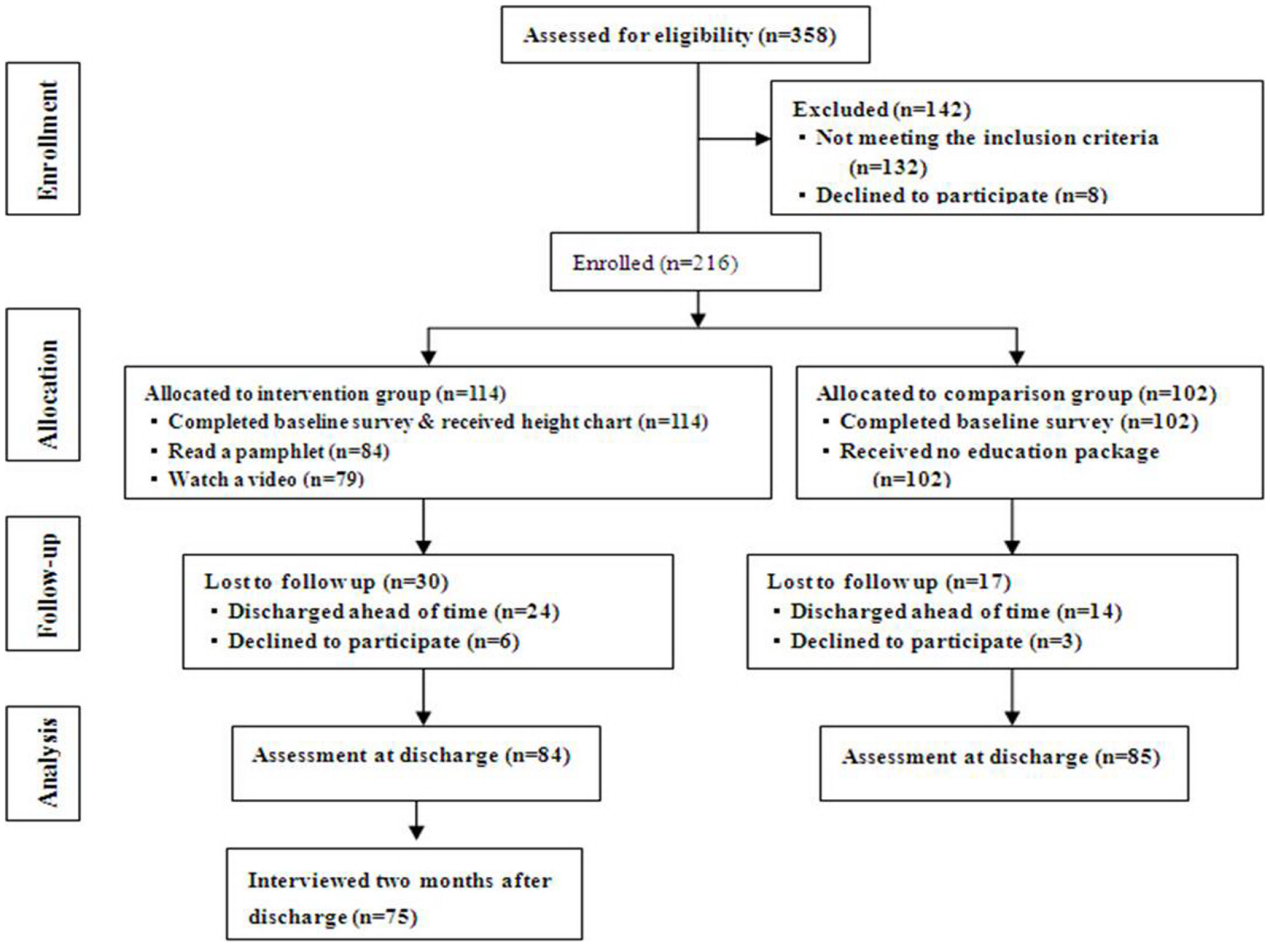
Chart of the Study Procedure.

Of the 358 birthing mothers approached in two hospitals during the study period, 216 mothers who owned a car in their household agreed to participate in the study. Of 142 mothers excluded from the study, 132 mothers did not have a car in their household, 2 mothers' children had died, and 8 mothers refused to participate in the program (Figure 1). Final enrollment of mothers consisted of 114 in the intervention group and 102 in the comparison group. Of these, 84 mothers in the intervention group and 85 mothers in comparison group completed follow-up surveys prior to discharge.

The demographics of participating mothers in the intervention group were similar to those in the comparison group (Table 1). Their average age ranged from 27 to 29 years old, with half of them having 1-5 years of driving experience. About 60% of the mothers received college or higher education. Over half of new mothers reported that they had seen a CSS before. Approximately, two-thirds of the mothers indicated that they would consider installing a CSS. The source of child safety restraint information was reportedly from networking, television, friend's use, or car retailers.

**Table 1:** Character of birthing mothers and their perceptions on CSS.

	Intervention group n(%)	Comparison group n(%)	χ^2^	P-value
	N = 114	N = 102		
**Age (mean ±Sd)**	29±4.3	27±3.9		
**Education level**			0.21	*P* = 0.94
Primary school	5(4.4)	4(3.9)		
Middle/high school	42(36.8)	35(34.3)		
College or higher	67(58.8)	63(61.8)		
**Driving experience**			3.34	*P* = 0.19
Less than 1 year	41(36.0)	25(24.5)		
1–5 years	47(41.2)	49(48.0)		
More than 6 years	26(22.8)	28(27.5)		
**Family monthly income**			0.90	*P* = 0.64
Less than 3000 Yuan	12(10.5)	7(6.9)		
3000–6000 Yuan	47(41.2)	44(43.1)		
More than 6000 Yuan	55(48.2)	51(50.0)		
**Ever heard of or seen CSS**			4.14	*P* = 0.25
Never heard or seen	14(12.3)	14(13.7)		
Heard but not seen	25(21.9)	34(33.3)		
Seen it	65(57.0)	47(46.6)		
Ever used	10(8.8)	7(6.9)		
**Where learned about CSS ^1^**			5.79	*P* = 0.22
Internet	44(38.6)	37(36.2)		
TV	43(37.7)	28(27.4)		
Friends	33(28.9)	30(29.4)		
Car store	11(9.6)	20(19.6)		
Others	21(18.4)	22(21.6)		
**Consider installing child safety seats when buy a car**				
Yes	77(67.5)	67(65.7)	0.08	*P* = 0.88
No	37(32.5)	35(34.3)		

Note: 1. Sum of percentage is over 100 due to multiple choices.

2. P-values are based on chi-square tests.

### Evaluation of intervention implementation

Among the 114 intervention mothers who completed the baseline and received the intervention, 84 (73.3%) read the pamphlets, and 79 (69.3%) watched the educational video. Over 90% (77 out of 84) of intervention mothers rated the educational intervention as helpful. Of 79 mothers who watched the educational video, 37.9% rated the video as very helpful, another 30.4% and 29.1% stated it was helpful and somewhat helpful, respectively. Of 84 mothers who read the pamphlet, 36.9% said it was very helpful, another 27.4% and 30.9% stated it was helpful and somewhat helpful, respectively (Table 2). Results from the subgroup analyses showed that mothers who rated the intervention as very helpful were more likely to purchase or use CSS following the intervention.

**Table 2:** Intervention mothers' evaluation of the intervention implemented in hospital at discharge (n = 84)^1^.

	Ratings			
	Very helpful N (%)	Helpful N (%)	Somewhat helpful N (%)	Not helpful N (%)
**Rating on helpfulness of the height chart for proper age of child safety seat use**				
Mothers who were introduced a height chart	62 (73.8)	14 (16.7)	5(5.9)	3(3.6)
Mothers who purchased CSS^2^ (n = 20)	12 (60.0)	5 (25.0)	3(15.0)	0
Mothers who used CSS^2^ (n = 9)	4 (44.4)	4 (44.4)	1 (11.2)	0
**Rating on helpfulness of the video for proper age of child safety seat use**				
Mothers who watched video^3^	30 (37.9)	24 (30.4)	23 (9.1)	2 (2.5)
Mothers who purchased CSS^2^ (n = 20)	10 (50.0)	5 (25.0)	4 (20.0)	1 (5.0)
Mothers who used CSS^2^ (n = 9)	5 (55.6)	2 (22.2)	2 (22.2)	0
**Rating on helpfulness of the pamphlets for proper age of child safety seat use**				
Mothers who read pamphlets	31 (36.9)	23 (27.4)	26 (30.9)	4 (4.7)
Mothers who purchased CSS^2^ (n = 20)	10 (50.0)	4 (20.0)	4 (20.0)	2 (10.0)
Mothers who used CSS^2^ (n = 9)	5 (55.6)	2 (22.2)	1 (11.1)	1 (11.1)

1. Though 114 mothers were offered the intervention, 84 of them were followed 2 months after discharge and reported their ratings.

2. Ratings of the subgroups of mothers who purchased or used CSS are presented.

3. The results were based on 79 mothers who watched the video.

### Differences in outcomes before and after the intervention

There was a significant difference in intervention mothers' awareness of and attitudes towards safe traveling practice pre- and post-intervention. Specifically, the percentage of intervention mothers who identified CSS as the safest seating practice increased from 52.6% to 86.9%, while the percentage among control mothers remained at approximately half in both the pre- and post-tests (Table 3). The percentage of intervention mothers who reported that CSS are necessary increased 10% post-intervention but did not change among the control group. Two months after discharge, the follow-up telephone interview found that there were 20 (26.7%) intervention families who purchased CSS for their babies following the intervention, with 1 infant carrier and 19 convertible seats. Nine out of 20 families reported that they had used it, which was 12% of the population. At baseline, only 8.8% of mothers reported use of CSS. Of 55 (73.3%) families that did not report using a CSS yet, 40 (72.7%) mothers said that they were planning to use one when their babies became 2 or 3 years old; 8 (14.5%) clearly stated they would not use it in the future, and 4 indicated they were not sure.

**Table 3:** Comparison of birthing mothers' child safety restraint knowledge before and after intervention.

	Intervention Mothers			Comparison Mothers		Difference^2^			
	Pre (n = 114)	Post (n = 84)	p-value^1^	Pre (n = 102)	Post (n = 85)	p-value^1^		p-value	
	N (%)	N (%)		N (%)	N (%)				
**Safest seating position for child**			0.18			0.69		0.003^4^	
Front seat next to driver	10(8.8)	7(8.3)		4(3.9)	4(4.7)				
Left or right rear seat	92(80.7)	74(88.1)		79(77.5)	61(71.8)				
Rear middle seat	12(10.5)	3(3.6)		19(18.6)	20(23.5)				
**Safest seating practice**			0.00			0.88		0.001**^4^**	
Hold in arm	13(11.4)	2(2.4)		7(6.9)	4(4.7)				
Rear seat with adult accompanying	29(25.4)	6(7.1)		34(33.3)	25(29.4)				
With seat belt in front seat	5(4.4)	1(1.2)		2(2.0)	3(3.5)				
With seat belt in rear seat	7(6.1)	2(2.4)		10(9.8)	9(10.5)				
Child safety seat or booster	60(52.6)	73(86.9)		49(48.0)	44(51.7)				
**Seat belt is better than CSS for child under 6**			0.25			0.96		0.27**^4^**	
No	89(78.1)	73(86.9)		76(74.5)	62(72.9)				
Yes	11(9.6)	6(7.1)		5(4.9)	4(4.7)				
Not sure	14(12.3)	5(5.9)		21(20.6)	19(22.4)				
**Necessity to use CSS**			0.03			0.73		0.27**^4^**	
No	5(4.3)	3(3.6)		6(5.9)	7(8.2)				
Not sure	15(13.2)	3(3.6)		24(23.5)	20(23.5)				
Yes	94(82.5)	78(92.8)		72(70.6)	58(68.3)				
**Proper age to use CSS ^3^**			0.95				0.67	0.03**^4^**	
0∼1 Y	54(47.3)	40(47.6)		48(47.1)	35(41.2)				
2∼5 Y	95(83.3)	75(89.3)		89(87.3)	68(80.0)				
6–13 Y	35(30.7)	25(29.7)		31(30.4)	30(35.3)				
**Classification of CSS ^3^**			-				-	0.00	
According to fixed way	-	33(39.3)		-	26(30.6)				
According to child age/weight	-	66(78.6)		-	33(38.8)				
Don't know	-	9(10.7)		-	26(30.6)				
**Consideration of future use**			-				-	0.75**^4^**	
Yes	-	41(48.8)		-	40(47.1)				
Maybe	-	42(50.0)		-	42(49.4)				
No	-	1(1.2)		-	3(3.5)				
**Reason for non-use of CSS^ c^**			-					0.24	
Insufficient knowledge	-	72(85.7)		**-**	66(77.6)		-		
Absence of laws	-	46(54.7)		**-**	39(45.9)				
High price	-	16(19.1)		**-**	19(22.3)				
Troubles with the usage	-	24(28.6)		**-**	38(44.7)				

Note: 1. There were 30 mothers in the intervention group and 17 mothers in the control group lost to follow up during the postpartum stay in the hospital. P-values were based on chi-square tests of the different pre- and post-interventions.

2. The differences were based on chi-square tests between intervention and comparison groups post- intervention. There were no significant differences between the intervention group and control group in the baseline pre survey.

3. Sum of percentage is over 100 due to multiple choices.

4 P-values were based on fisher exact tests.

### Differences in outcomes between intervention and comparison groups

There was a significant difference in the birthing mothers' child safety restraint awareness and attitudes between the intervention group and the comparison group after the intervention (Table 3). Specifically, nearly 93% of the intervention mothers reported that it was necessary for their child to use CCS while traveling in a car, compared to 68.2% of comparison mothers (p<.001). Nearly 87% of the new mothers in the intervention group reported that children should be placed in the CSS, compared to 51.7% of comparison mothers. In both the intervention and comparison groups, over 80% of mothers identified the ages of 2 through 5 as needing CSS, but fewer than 50% of both groups identified infants as needing CSS, suggesting that the program was not effective in teaching which age groups need CSS. Regarding safe practice for children under 6, we found that, compared to intervention mothers, a relatively higher percentage of mothers in the control group were still not sure whether CSS was better than a seat belt. The main reasons for not using a CSS were cited for both intervention and control mother as insufficient knowledge, followed by absence of laws, and/or troubles with usage.

## Discussion

This study evaluates a hospital-based child safety restraint educational intervention to birthing mothers during their 4- to 6-day hospital stay after delivery. The results showed that the intervention had an effect on birthing mothers' increased awareness and potential intention of CSS use. Specifically, over 90% of the birthing mothers in the intervention group found the intervention to be helpful to some extent, suggesting that the intervention was well received by the participating mothers. A significantly higher percentage of mothers in the intervention than the comparison group reported that CSS are necessary and are the safest seating practice. Nearly 20% of the intervention mothers actually purchased CSS for their babies after discharge. These findings have demonstrated some short-term effectiveness of the program and support future implementation of a hospital-based CSS educational intervention to birthing families in China.

Results indicate, however, that mothers lack knowledge about the optimal use of child safety seats with infants. Nearly twice as many mothers identified the ages of 2 to 5 as essential for CSS use as for child under 2 years old, and this was true for both the pre- and post-intervention periods and among both intervention and comparison group mothers. Over one quarter (26.7%) of the intervention mothers reported current use of a CSS, and over half (53.3%) reported that they planned to use one when their child is 2 or 3 years old. This evaluation clearly shows that while the program helped increased general need for the use of safety seats, further efforts are needed to address specific age-related needs and promote car seat use among infants and children older than five.

In contrast to the US, where child safety restraint laws were introduced [16]-[19] two decades ago, there is currently no compulsory legislation on child safety restraint in China. With few educational programs on child unintentional injury prevention, parents lack knowledge of child safety restraint use and proper behavior for safe child transportation. Our success in implementing the hospital-based CSS program was partly attributed to our tailoring of the child safety restraint education program in the hospital setting for a specific population of birthing mothers [20].Our study identified some barriers with the use of CSS, including knowledge of the proper age, preference for holding infants in a parent's lap, and reports that seats are difficult to work with. Mothers also reported not using seats because there is no legislation. Although 20% of the intervention mothers purchased a CSS, only half of those were actually using it. The findings from this study call for more CSS educational programs to increase knowledge and overcome barriers in attitudes about safety seats. These educational programs should be implemented to augment programs to increase availability and affordability of CSS, and policies that require their use [12], [13].

This is an education-only program providing information about child safety restraint and the importance of safe traveling practices to birthing mothers. Previous studies suggest that knowledge loss may occur over time without reinforcement and that knowledge alone may not be sufficient in leading to behavior change [21]. This may in part explain why the majority of parents agreed that CSS is necessary for children when traveling, yet only 20 out of 84 of families purchased the CSS after discharge and only 9 families actually used it. To successfully increase infant and child restraint seat use, many other evidence-based strategies, in addition to educational programs [22], could be considered. For example, in the U.S., multiple hospital-based interventions strategies, such as formal infant CSS discharge policies, CSS education and CSS loan or giveaway programs, have been used at hospital discharge and have demonstrated success in increasing CSS use [23], [24], [25], [26].

This result of self-reported CSS use confirmed our earlier observation that only 1.2% of children were restrained in CSS among the 3,333 children traveling in cars [4]. Our findings indicated that it is difficult to change CSS use behavior and we believe that this low rate of use is the combination of inadequate CSS promotion, poor safety awareness, social norms, and lack of a mandatory requirement for child safety restraint in present day China. Our findings, along with those of others [27], [28] may have some implications. Few newborn mothers are receiving adequate education on safe child passenger traveling; lack of education and law might result in widespread non-use of CSS in the proper ages. CSS producers or those who are interested in children's vehicle safety restraints should explore a proper car seat rental scheme as advocated in hospital and kindergarten settings. We recommend that the hospital, school, manufacturers, and government take comprehensive efforts on safe traveling for the prevention of injury in children (Table 4).

**Table 4:** Comprehensive efforts on safe traveling mode of traffic injury prevention.

	Hospital	School	Manufacturers	Government
Promotion of CSS used in child traveling	Design baby safe traveling class before delivery; Physician's advice and suggestion on CSS use before dischargeAllow CSS rental scheme provided by manufacturers.	Teach children knowledge about safe seating in car.	Produce safe device and advertise the necessity of using CSS (CSS manufacturer); Providing CSS choice when selling a car (car manufacturer).	Support education by financial administrative means; formulate legislation related to CSS use.

### Limitations

First, since our sample population was a sample of birthing mothers in the hospital who own a car, the results of this study could not be generalized to all birthing mothers. Second, during the limited time of a hospital stay, mothers' interest in infant feeding, bathing, and immunizations often outweigh safe traveling education. Thus, we were not able to recruit or follow-up with all enrolled mothers. Third, this study was not able to provide free or rental CSS, and it was also not possible to demonstrate the correct use of CSS in real life in the obstetric department, which may influence mothers' use of CSS after discharge. Also, it was unfortunate that we failed to follow-up the comparison group to determine actual use of CSS. Finally, a short term effect of the program was measured in this study, but a long term impact on attitudes was not yet captured.

## Conclusion

This study evaluates a hospital-based education intervention to promote child safety restraint use, especially in infants. The program improved the birthing mothers' knowledge and awareness, which could drive them to prepare CSS for their babies. This study has implications for future comprehensive intervention strategies that address specific age-related needs and promote car seat use among infants and children.
